# Structural characteristics of potential novel virulence factors, the host cytokine binding proteins of *Aggregatibacter actinomycetemcomitans*

**DOI:** 10.1080/20002297.2017.1325205

**Published:** 2017-05-30

**Authors:** Riikka Ihalin

**Affiliations:** ^a^ Department of Biochemistry, University of Turku, Turku, Finland

## Abstract

Various gram-negative pathogens possess secreted or outer membrane proteins (OMPs) which interact with host cytokines. Of periodontal pathogens only *Aggregatibacter actinomycetemcomitans* (*Aa)* has been shown to sequester and uptake cytokines. We have identified two (OMPs) of *Aa*, bacterial interleukin receptor I (BilRI) and OM secretin channel (OMS), which interact with several cytokines, the interaction with IL-8 being the strongest. The NMR studies confirmed that BilRI is intrinsically disordered, *i.e*. it does not have stabile fold without ligand. The structure of the extramembranous domains of OMS (emOMS) solved with X-ray crystallography showed that the C-terminal domain of emOMS displayed a type I KH domain fold. Crosslinking experiments indicate that one lysine of this domain is located in the IL-8 interaction site. *Neisseria meningitides* PilQ (NmPilQ) has role in the uptake of IL-8. Although the part of the NmPilQ which interacts with IL-8 is unidentified, the N1-domain of NmPilQ shows sequence homology and adopts the same fold as the C-terminal domain of emOMS. Thus the type I KH domain fold might be involved in the cytokine interaction in both these bacterial proteins. It would be interesting to learn if type I KH domains can be found from other periodontal pathogens than *Aa*.

